# Grand challenge of maintaining meaningful communication in dementia care

**DOI:** 10.3389/frdem.2023.1137897

**Published:** 2023-03-03

**Authors:** Wendy Moyle

**Affiliations:** ^1^Menzies Health Institute Queensland, Griffith Health, Griffith University, Brisbane, QLD, Australia; ^2^School of Nursing and Midwifery, Griffith Health, Griffith University, Brisbane, QLD, Australia

**Keywords:** communication, dementia, digital sources, social robots, communication device

## 1. Introduction

Alongside the aging of the world population, the number of people with dementia is growing. Approximately 55 million people currently live with dementia globally, with a prediction that this number will increase to 79 million in 2030 (World Health Organization, 2022). Many people with dementia end their lives in a residential aged care facility (RACF) as their cognitive and physical status deteriorates. Around 68% of aged care residents in Australia have moderate to severe cognitive impairment [Australian Institute of Health and Welfare (AIHW), 2022]. In the US (Freedman et al., 2021) and the UK (Alzheimer's Society UK, 2022), around 70% of people aged 70 plus years living in nursing homes have dementia.

Physical (e.g., muscle weakness, weight loss, changes in sleeping, and eating habits) and cognitive (e.g., problems with thinking, remembering, reasoning, language, and visual perception) are changes associated with dementia and these increase the likelihood of dementia-related agitation, called behavioral and psychological symptoms of dementia (BPSD) (Cummings et al., 2015). These behaviors reduce quality of life (Klapwijk et al., 2016) and increase the use of hospital and health services (Burley et al., 2020). Global spending on dementia care is substantial and estimated to be US $594 billion and to reach US $1.6 trillion by 2050 (Pedroza et al., 2022) alongside a growth in the aging population. In addition, the costs of hospital care for people with dementia are higher than those without dementia [Australian Institute of Health and Welfare (AIHW), 2022]. In community care, the high care costs are associated with the burden of care experienced by families and carers. This burden of care is likely to increase as the number of informal carers continues to increase, with an estimated 11 million American family and friends providing unpaid care for people with dementia (Alzheimer's Association, 2022).

## 2. The challenge of deteriorating communication

Various challenges are associated with the care of people with dementia, particularly as dementia is associated with high rates of unmet needs. For example, social isolation is an enormous challenge for people with dementia and is associated with numerous adverse outcomes, including loneliness (Rippon et al., 2020). However, one of the most significant challenges this population and their family carers experience early in the condition is the insidious onset of deteriorating communication (Saunders et al., 2011), which reduces the opportunities to engage in meaningful communication (Moyle et al., 2014).

Language difficulties are challenging for most people with dementia. The cognitive changes associated with dementia can make it demanding for the person with dementia to follow and participate in conversations. For example, a decline in memory, attention, executive functioning, and language processing can result in the individual having trouble finding the right word, especially when naming people or objects. They may repeat words and phrases or replace words with inappropriate ones making conversation challenging. People with Alzheimer's disease, the most common form of dementia, have difficulties with comprehension, fluency, verbal feedback, and word production (Banovic et al., 2018). These types of communication challenges can cause frustration, and as a result, the person with dementia may show exasperation and express this through inappropriate behaviors. In addition, the lack of social stimulation in RACFs adds to the person with dementia's feelings of social isolation and loneliness. The maintenance of communication is essential between families and people with dementia to enable individualized care provision and meaningful connection, which is intrinsic to mental health and wellbeing.

When people with dementia are placed into a RACF, this can relieve family carers of the burden of care if they take the opportunity to reduce their time spent with the person with dementia. However, moving into a RACF may also add to geographical separation and an additional travel burden if they wish to visit, reducing opportunities to connect, engage and communicate. Communication is essential in the care of a person with dementia as communication activities can improve wellbeing (Knebel et al., 2016). Deteriorating communication can decrease the quality of life for the person with dementia and reduce the opportunity for the person to contribute to activities around them. Reduced opportunities for stimulation can adversely affect mood and increase agitation (Moyle et al., 2016).

COVID-19 has further reduced opportunities for people living with dementia in RACFs to engage with families and others. The indirect effects of COVID-19 include increases in mental illness, such as loneliness and depression [Australian Institute of Health and Welfare (AIHW), 2022]. Furthermore, visiting bans during COVID-19 have meant residents have had to rely on staff for communication and to use the telephone and digital aids to communicate with family and friends (Lion et al., 2022). However, these activities are complicated by staff having limited language and technical skills (Eriksson and Hjelm, 2022).

Although dementia affects the communication of the person with dementia, there is a growing need to support the training of RACF care staff to improve communication care. RACF staff are frequently challenged by large workloads, so they tend to focus on task-orientated care such as hygiene and nutrition. Health and care professionals currently receive little communication training. Improving staff and family awareness of the opportunities offered by communication can have a positive impact (Nguyen et al., 2019). For example, offering the opportunity to support family carers can improve outcomes for the person with dementia and reduce BPSD. In addition, training may improve staff attitudes and cultural insensitivity that can inhibit communication for people with dementia (Brooks et al., 2019).

It is imperative to approach a person with dementia correctly when communicating. Jootun and McGhee (2011) suggest that when communicating with a person with dementia always face the person and speak directly to them. Gain the person's attention before starting a conversation. Use simple language and speak slowly. Give the person time to process the information and to respond. Finally, use facial expressions to help the person understand what is being communicated. For example, smile when the message is pleasurable.

## 3. Emerging opportunities: Communication devices

Communication support is anything that improves access to communication, activities, or events (Fried-Oken et al., 2015). This could be modifications to the environment or supportive devices. For example, using communication devices may support people with dementia and their carers to communicate more effectively by increasing the frequency and duration of conversations (Ekström et al., 2017). Such devices are more likely to be effective and feasible if they have been co-designed with people with dementia and their family and formal carers. However, such devices are in their infancy and may require further development and testing to be effective in this population.

Importantly, is a need for staff and family training in communicating and connecting when a person has dementia. Currently, there is minimal staff available in RACF, which reduces the opportunity to support communication between families and residents. Therefore, when using communication devices, these need to be self-supporting or allow easy access for people with dementia and their families.

In recent years attention has been given to word games to help overcome memory problems and word-finding difficulties. Tools to help communication have included photo albums, digital storybooks (Da Silva et al., 2022), and digital tablets (Samuelsson and Ekström, 2019). Digital devices that help to create reminiscence activities, such as music and photos, have encouraged conversations between people with dementia and their families (Dassa, 2018; Garlinghouse et al., 2018). In addition to digital devices, social robots have been found to engage people with dementia in conversation with the robot or/and family or facilitator (Liang et al., 2017; Moyle et al., 2019a). For example, animal robots such as PARO can engage people with dementia to communicate with the robot without concerns about hygiene or being bitten (Moyle et al., 2017b). Animal type robots are well-accepted in dementia care with dogs, cats and the PARO robot being the most popular.

During COVID, many RACF staff reached for tablets/iPads as digital communication support for people living with dementia. Digital tablets are useful as reminiscence aids allowing a display of tools and encouraging communication. However, as a tool to engage people with dementia in conversations, they offer possibilities and potential difficulties in their use. More significant problems relate to tablets not supporting communicative initiatives (Ekström et al., 2017) and people with dementia requiring considerable support to use a digital communication device (Moyle et al., 2020a). Moyle et al. (2020a) identified four themes around the use of iPads with older residents in nursing homes. One positive theme included that video conferencing was a positive way for residents to communicate with family and friends. However, three negative themes included, (i) videoconferencing on iPads was inhibited by cognitive decline and physical frailty, (ii) videoconferencing required practice and staff assistance, and (iii) concerns were held about cyber security and privacy, therefore inhibiting the use of iPads by older residents living in RACFs.

On the other hand, device such as telepresence robots (e.g., double 3, https://www.doublerobotics.com/) can enhance opportunities for family and friends to connect with a person with dementia, even from afar (Moyle et al., 2014, 2020b). These robots allow the operator (family or friend) to see the person with dementia through a two-way screen. This can comfort families and friends when seeing a person looking clean and comfortable. The telepresence robots eliminate the need for the resident to manage the software and hardware as the remote operator manages this. They are a freestanding, wheel-based videoconferencing system with two cameras, a pan-tilt LCD screen, self-driving sensors and microphones. The remote operator and the telepresence robot are connected *via* software and the internet. The telepresence robot is mobile, allowing it to move freely throughout the facility, and providing visual cues to enrich conversations. For example, the opportunity to view and talk about new plants in the garden, artwork in the building or staff offers a richness that is unavailable *via* a telephone call, alleviating stress and feelings of disconnection during visits. Importantly, family and friends can manage the robot from afar, reducing the need for staff involvement. Unlike an iPhone or iPad, residents do not need to manage camera placement. Research conducted by our team has shown the benefit of this technology in engaging people living with dementia in RACFs with family and friends (Moyle et al., 2014, 2017a, 2019b, 2020b).

## 4. Concluding comments

While there is a lack of research on communication devices and people with dementia, the available studies suggest promising opportunities for such technologies in engaging communication (Hoel et al., 2021). Due to the effect of dementia on communication and its impact on the person and their family and friends, there is a need to tailor opportunities to improve quality of life. To increase meaningful communication opportunities for people with dementia in RACFs, a focus on effective communication devices such as telepresence robots that will engage family and friends is needed. Ongoing co-design of new devices for people with dementia and families and training of staff and family is needed to enhance communication opportunities. Furthermore, people with dementia and their families need to be involved in the evaluation, usefulness, acceptability, and effectiveness of such technologies. Finally, guidelines for using such devices must be developed, considering budgets and the effectiveness of such products (Moyle, 2019).

## Author contributions

The author confirms being the sole contributor of this work and has approved it for publication.
